# A Role for Zinc in Plant Defense Against Pathogens and Herbivores

**DOI:** 10.3389/fpls.2019.01171

**Published:** 2019-10-04

**Authors:** Catalina Cabot, Soledad Martos, Mercè Llugany, Berta Gallego, Roser Tolrà, Charlotte Poschenrieder

**Affiliations:** ^1^Departament of Biology, Universitat de les Illes Balears, Palma, Spain; ^2^Plant Physiology Laboratory, Bioscience Faculty, Universitat Autònoma de Barcelona, Barcelona, Spain

**Keywords:** zinc deprivation, zinc toxicity, zinc hyperaccumulation, Zn-triggered organic defenses, plant enemies

## Abstract

Pests and diseases pose a threat to food security, which is nowadays aggravated by climate change and globalization. In this context, agricultural policies demand innovative approaches to more effectively manage resources and overcome the ecological issues raised by intensive farming. Optimization of plant mineral nutrition is a sustainable approach to ameliorate crop health and yield. Zinc is a micronutrient essential for all living organisms with a key role in growth, development, and defense. Competition for Zn affects the outcome of the host–attacker interaction in both plant and animal systems. In this review, we provide a clear framework of the different strategies involving low and high Zn concentrations launched by plants to fight their enemies. After briefly introducing the most relevant macro- and micronutrients for plant defense, the functions of Zn in plant protection are summarized with special emphasis on superoxide dismutases (SODs) and zinc finger proteins. Following, we cover recent meaningful studies identifying Zn-related passive and active mechanisms for plant protection. Finally, Zn-based strategies evolved by pathogens and pests to counteract plant defenses are discussed.

## Mineral Nutrients in Plant Defense

Mineral nutrients are directly involved in plant protection as structural components and metabolic regulators (Huber, 1980). As a first line of defense, the nutritional status can determine plants’ susceptibility to pests and pathogens (Walters and Bingham, 2007; Marschner and Marschner, 2012). Essential and beneficial elements affect plant health both directly, by activating enzymes that produce defense metabolites (callose, glucosinolates, lignin, phenols, and phytoalexins), and indirectly, by altering root exudates, rhizosphere pH, and microbial activity (Datnoff et al., 2007). In addition to chemical and/or biochemical factors, plant protection strategies include physical (shape, surface properties, hairs, color, etc.) and mechanical (fibers, silicon) properties (Marschner, 1995); all these features are also influenced by mineral nutrients.

The nutritional status of the host plant affects the colonization success by pests and pathogens. However, it is not possible, either globally or individually, to generalize the effects of nutrients for all plant–pest/pathogen systems (Huber, 1980).

Many studies have focused on the relationship between macronutrients and plant pests and diseases, and several sound reviews link both topics. Most attention has centered on the effects of N, P, and K due to their reduced availability in many soils and their elevated plant demand (Huber, 1980; Walters and Bingham, 2007; Amtmann et al., 2008). However, inconsistent results of macronutrient fertilization and damage by insect herbivory have been reported (Aqueel and Leather, 2011; Chesnais et al., 2016). Furthermore, silicon has deserved special attention, primarily on account of its structural role in the reinforcement of cell walls, and more recently as an activator of defense genes (Fauteux et al., 2005; Cabot et al., 2013; Van Bockhaven et al., 2013) and pest protection (He et al., 2015).

Micronutrient roles in plant defense are predominantly documented for Mn, Cu, Fe, and Zn (Graham and Webb, 1991; Dordas, 2008; Fones and Preston, 2013). Manganese participates in the production of phenolic compounds and other plant defense mechanisms (Fernando et al., 2009), while Ni is involved in the plant antioxidant system and in the plant response to stress (Fabiano et al., 2015). Best studied are the pathogenesis-related mechanisms based on the Fe redistribution at the cellular level (Greenshields et al., 2007; Liu et al., 2007). Zinc seems to be a major player in both animal (Hojyo and Fukada, 2016) and plant immune responses (Shirasu et al., 1999; Gupta et al., 2012a). However, so far, no general model has been proposed for Zn, despite the fact that several processes involving this element have been analyzed in different pathosystems. Furthermore, most of these studies have focused on metal hyperaccumulating species that absorb and accumulate a wide range of elements, including micronutrients like Mn, Fe, Ni, and Zn, or other trace elements like Cd, Se, or As. The high tissue concentrations of these elements may protect plants against pests and diseases (Poschenrieder et al., 2006) (see below).

This review complies the still scant available information characterizing the Zn-related strategies employed by plants to fight their attackers. The generated overview will hopefully permit the reader to see the state of the art of this topic, as well as the existing gaps, where current knowledge established in plant systems is derived either from Fe research or taken from animal systems.

## Defense-Related Functions of Zinc in Plants

Zinc is a catalytic and structural protein cofactor in hundreds of enzymes (Hambidge et al., 2000) and has key structural functions in the protein domains that interact with other molecules. The “Zn finger” proteins mediate DNA binding of transcription factors and protein–protein interactions (Sinclair and Kramer, 2012). Zn-binding sites can now be predicted from sequenced metal-binding motifs using bioinformatic approaches. Based on these findings, the Zn proteome may represent about 9% of the entire proteome in eukaryotes and from 5% to 6% in prokaryotes (Andreini et al., 2009).

Both Zn excess and Zn deficiency cause Zn to become prooxidant (Kinraide et al., 2011). Therefore, in all organisms, the cellular concentration and compartmentation of mobile Zn is tightly controlled by different sets of proteins responsible for sensing, transporting, buffering, storing, and releasing Zn. An outstanding reason for the requirement of a strict control of Zn homeostasis comes from studies conducted in animal systems demonstrating that Zn is an intracellular second messenger in several signaling pathways (Yamasaki et al., 2007); therefore, this element plays key roles in the organism’s metallo-neurochemistry. Zinc acts as a neuromodulator in the central nervous system (Goldberg and Lippard, 2018), and Zn transporter ZnT3 is responsible for Zn concentration in synaptic vesicles of certain glutamatergic neurons (McAllister and Dyck, 2017). While glutamate has a recognized function in plant signaling (Forde and Lea, 2007), a connection to Zn in this process has not yet been established in plants.

Zinc plays a pivotal function in the plant response to pests and diseases. Nonetheless, Zn defense-related mechanisms in plants greatly vary. The outcomes of plant–pest/pathogen interactions differ, depending on the effectivity of the Zn-related responses in limiting the invader’s attack as well as on the enemy’s ability to circumvent the plant defenses, in addition to other environmental conditions that can favor either host or invader. Several studies have shown that, in most cases, Zn fertilization decreased plant symptoms (Grewal et al., 1996; Li et al., 2016; Machado et al., 2018). However, a protective Zn concentration against certain pathogens can also induce a higher susceptibility to another pathogen on the same plant (Helfenstein et al., 2015) (see below).

Zn proteins play a dual role in plant defense, with potential to simultaneously aid and abet the plant and its invaders. **Table 1** provides evidence of some of the described connections of Zn proteins with the plant defense and/or the invader virulence. The most commonly assigned functions of Zn proteins are listed in this table, along with examples relating a specific protein function with their respective defense mechanisms. The table denotes that certain similar Zn protein-based mechanisms have been described for both plant defense factors and the pathogenicity factors of their invaders. In the reported cases, the described mechanism is either exclusively mediated through Zn or Zn is mentioned to be involved. The results of these studies summarized in **Table 1** globally address an effective plant defense/invader attack with increased gene expression or enhanced activity of the referred protein function. It should be underscored that the described strategies of defense are not exclusively assigned to Zn proteins but also combined with other responses. Among those, two broad-spectrum responses involved in plant–pest/pathogen interactions are tightly related to Zn: oxidative stress and regulation of Zn finger proteins, both of which are more extensively discussed in the following subsections.

**Table 1:** Most common assigned functions of Zn proteins with examples relating a specific protein function with defense mechanisms. Similar Zn protein-based mechanisms have been described for the plant defense factors against pathogens (P) or phytophagous insects (I).

	Role in defense of plants challenged by pathogen/ insect	Role in pathogen/herbivore virulence
Alcohol dehydrogenase (AD)	(P) Up-regulation of zinc-binding AD in pathogen-inoculated plants. Mechanism display in resistant cultivars (Kumar et al., 2016). (I) Cinnamyl AD (CAD) increased activity induce reduced fitness pest (Cipollini, 1997). / CAD higher activity related to resistance after pest infestation (Shivashankar et al., 2015).	–
Carbonic anhydrase (CA)	(P) CA function of salicylic acid-binding protein required for pathogen infection (Wang et al., 2009). / CA-silenced plants more susceptible to pathogen spreading (Restrepo et al., 2005). (I) -	–
α-Mannosidase (Ma)	–	(P) Effector with Ma function required for pathogenesis of pathogenic fungus (Martinez-Cruz et al., 2018). (I) -
Metallothionein (MT)	(P) MT1 highly expressed during fungal infection (Kim et al., 2001). / High constitutive expression of certain MT genes in resistant plant variety (Degenhardt et al., 2005). (I) MT involved in the plant resistance to pest-fungal interaction (Calic et al., 2017).	(P) MT1 with very high affinity for Zn essential for fungal pathogenicity involved in penetration of leaf surfaces (Tucker et al., 2004). / Putative metallothioneins strongly up-regulated during fungal parasitic growth (Jakupovic et al., 2006). (I) Enhanced MT levels in specialized (Liu et al., 2014) and polyfagus pests (Shu et al., 2012) after ingestion of heavy-metal enriched diets.
Superoxide-dismutase (SOD)	(P) SOD-transformed lines showed increased resistance to pathogenic bacteria (Kim et al., 2007). / ROS detoxification proteins highly expressed in pathogen-infected resistance cultivars (Wongpia and Lomthaisong, 2010). / Enhanced SOD levels, among other enzymes, in the highly resistant plant species to fungal pathogen (Subramanian et al., 2005). / Elevated SOD activity in virus-infected leaves (El-Moshaty et al., 1993). (I) Increased SOD activity after insect damage (Chang et al., 2012). / SOD activity negatively correlated with foliar damage after pest attack (Khederi et al., 2018).	(P) Pathogenicity factor confirmed for Zn-only SOD (Liu et al., 2016). / Cu-Zn SODs conserved in wide range of plant pathogens (Fones and Preston, 2012). (I) SOD crucial to control prooxidant activity of allelochemicals in plant pests (Pritsos et al., 1991). / SOD crucial to prevent the first step of the free-radical cascade of oxygen in herbivorous insects (Ahmad, 1992).
Zn finger (Znf)	(P) Znf gene key in the R-gene-specific resistance of plants to pathogens (Liu et al., 2002; Tornero et al., 2002; Wang et al., 2017). / Znf transcription factor (TF) contributes to enhance disease resistance (Kim et al., 2004). (I) Insect infestation induces Znf TF synthesis (Lawrence et al., 2014) / Znf TF play significant role in the resistance to insects (Schweizer et al., 2013).	(P) Znf TF of fungal pathogen involved in phytoalexin detoxification (Kettle et al., 2016). (I) -

“–“ means Not yet described.

### Superoxide Dismutases (Sods)

Zinc affects plant–pathogen interactions *via* its key role in the activation/stabilization of metalloenzymes (Fones and Preston, 2012). A common component in the plant responses to stress conditions caused by insufficient Zn availability and/or pathogen attack is the plant’s capacity to overcome oxidative stress. Under Zn-deficient conditions, reactive oxygen species (ROS) are considered to be the primary factor responsible for plant growth inhibition (Cakmak, 2000). Additionally, changes in the antioxidant capacity and increased ROS formation have also been reported in plants in response to excess Zn (Jain et al., 2010; Feigl et al., 2015). On the other hand, the immune system uses ROS to fight pathogens directly by causing oxidative damage or indirectly by triggering different non-oxidative mechanisms, i.e., pattern recognition and receptor signaling. After plant recognition of pathogen attack, a rapid oxidative burst of oxygen radicals is needed in many cases to trigger plant defense mechanisms, i.e., the hypersensitive response and systemic acquired resistance (SAR) (Sutherland, 1991; Lehman et al., 2015). Oxygen radicals are controlled by the activity of antioxidant enzymes, and among them, SODs catalyze the conversion of superoxide radicals to hydrogen peroxide, which is involved in abiotic and biotic stress signaling (Miller et al., 2008). Cu/Zn-SOD activity is commonly increased in herbivore/pathogen-challenged plants (Fodor et al., 1997; Montalbini and Buonaurio, 1986; Zacheo and Bleve-Zacheo, 1988; Deepak et al., 2006), yet its activity decreased in Zn-deficient plants (see below).

### Zinc Finger Proteins

In addition to their role in plant growth and development, zinc finger proteins regulate plant responses to biotic stress conditions (Noman et al., 2019b). With a broad spectrum of structures and functions, these proteins are defined as those with a small, freely folded functional domain that requires one or more zinc ions to stabilize its structure (Laity et al., 2001). Zinc finger binding domains are present in the well-known plant resistance proteins NBS-LRRs (nucleotide binding sites-leucine rich) that are involved in the effector-triggered immune response (Gupta et al., 2012a). The authors of this study analyzed 70 plant disease-resistance proteins from different crops. Of these proteins, 37% contain zinc finger domains, which suggests a major role for this protein class in the host’s resistance to pathogens. One of the R-genes with a zinc finger domain studied was Pi54, which confers durable resistance against the fungus *Magnaporthe oryzae*. In a transgenic line of rice containing the Pi54 gene, the up-regulation of defense response genes (callose, laccase, PAL, and peroxidase) and genes related to transcription factors (NAC6, Dof zinc finger, MAD box, bZIP, and WRKY) was observed (Gupta et al., 2012b). Additionally, RAR1, a zinc binding protein of wheat, confers resistance against the stripe rust pathogen through salicylic acid (SA)-mediated oxidative burst and hypersensitive response (Wang et al., 2017). Insect infestation has been related to the up-regulation of two zinc finger transcription factors in potato (Lawrence et al., 2014). Some members of the family of transcription regulators cysteine2/histidine2-type zinc finger proteins (ZAT) play key roles in ROS signaling in the response of plants to biotic and abiotic stresses (Miller et al., 2008). A member of this family, AtZAT6, participates in plant development and positively modulates the *Arabidopsis* response to pathogen infection by activating the expression of SA-related genes such as PATHOGENESIS-RELATED GENE1 [*PR1*], *PR2*, and *PR5*.

**Figure 1:**
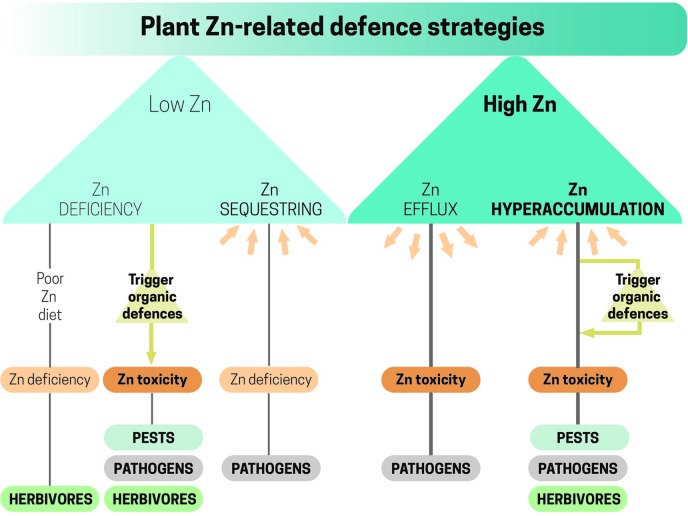
Low- and high-Zn conditions created by plants to confront pests, pathogens, and herbivores. Low-Zn conditions when resulted from a poor Zn diet can trigger the synthesis of organic defenses against a wide variety of plant enemies. Active Zn-sequestering/efflux from/to the extracellular media reduces/increases Zn availability, causing deficiency/toxicity to pathogens. The presence of high Zn concentrations in the above-ground parts of Zn hyperaccumulating species causes Zn toxicity to plant attackers.

In plant and animal systems, direct roles of Zn to control pests and pathogens include mechanisms to produce either low- or high-Zn scenarios (**Figure 1**). Host-imposed Zn starvation by sequestering Zn from the infection site in a biologically inactive form is a common strategy deployed by the immune system in animals to fight pathogens (Vignesh et al., 2013). Zinc removal strategies from the media to prevent microbial growth have been less documented in plants, yet most of the research on this topic has focused on Fe (Fones and Preston, 2013). On the other hand, hosts may actively deploy high Zn concentrations to control pathogen proliferation. Such active mechanisms have mostly been reported in animal systems (McDevitt et al., 2011), while few studies have been conducted in plants so far (Stolpe et al., 2017). Most of the research in plants concerning the potential protective role of high Zn concentrations has focused on Zn hyperaccumulating species. High Zn concentrations could protect plants from invaders by direct Zn toxicity and by enhancing Zn-triggered organic defenses (Poschenrieder et al., 2006; Fones and Preston, 2013).

In the following sections, plant strategies to fight pests and diseases related to low and high Zn concentrations as well as mechanisms evolved by pathogens and pests to counteract Zn-related plant defenses will be discussed.

## Defense Mechanisms Against Biotic Stress Under Low-Zinc Conditions

### Role of Zn Deficiency in Plant Defense

In general, Zn-deficient plants are more susceptible to diseases (Marschner, 1995; Grewal et al., 1996; Streeter et al., 2001; Helfenstein et al., 2015). Most studies looking for a potential relationship between plant Zn status and disease severity have reported an ameliorated response to diseases caused by fungi in plants supplemented with Zn (Grewal, 2001; Simoglou and Dordas, 2006; Huber and Haneklaus, 2007; Khoshgoftarmanesh et al., 2010). For example, a study conducted on the effect of different pathogens in soybeans with different Zn treatments showed that plants grown with either normal or high Zn fertilization had fewer positive counts for bacterial pustules caused by *Xanthomonas axonopodis* pv. *glycines* and less lesion area affected by the necrotrophic fungus *Sclerotinia sclerotiorum* than plants grown with low Zn (Helfenstein et al., 2015).

Along this line, Zn-efficient genotypes have been found to be more disease resistant, showing a positive relationship between increased plant growth and reduced pathogen susceptibility (Graham and Webb, 1991). Although the response of plants to Zn deficiency has been extensively studied, the different components and mechanisms involved in the efficient use of Zn remain unclear (Hacisalihoglu and Kochian, 2003). The oxidative damage due to ROS production is reduced in Zn efficient genotypes (Rose et al., 2012). Moreover, supplemental Zn has been found to increase SOD activity in plants under abiotic stress (Noman et al., 2018).

Recently, important advances on microRNA (miRNA) functions have been achieved, which would contribute to clarifying the mechanisms involved in the response of plants to pests and pathogens under Zn scarcity. In addition to plant growth and development, miRNAs modulate plant responses to stress conditions (Noman et al., 2017b). The ROS-quenching metalloenzyme Cu/Zn-SOD is post-transcriptionally regulated by a miRNA; specifically, the miR398 targets isoforms CSD1 and CSD2, which are located at the cytosol and chloroplast, respectively (Jones-Rhoades et al., 2006). The down-regulation of miR398 induces the CSD1 gene expression, a mechanism triggered by bacterial infection (*Pseudomonas syringae*) in *Arabidopsis* (Sunkar et al., 2006). However, miR398 was up-regulated under low Cu^2+^ concentrations. Although the effect of Zn starvation on miR398 has not been reported, the up-regulation response found for Cu could also be associated to Zn, as the protein is cofactored by both elements. Nonetheless, further studies are needed to clearly establish the relationship between Zn deficiency and pathogen susceptibility.

Reduced protein synthesis is one effect of Zn deficiency that leads to a higher accumulation of amino acids. This increase has been related to higher incidences of sucking insects present on the plant (Fageria et al., 2002). Similar results were obtained for Zn-deficient soybean plants that showed increased aphid colonization compared to plants grown at physiologically sufficient Zn concentrations (Helfenstein et al., 2015). Contrastingly, in the same study, the biotrophic obligate pathogen that causes soybean rust, *Phakopsora pachyrhizi*, had a lesser rate of spread in deficient plants. The authors concluded that one single element can differentially affect the response of a specific host to aphids and pathogens. These results revealed the lack of a Zn-specific requirement to fight pests and pathogens, which is a limiting factor for the use of micronutrient fertilization to manage diseases.

Some of the mechanisms involved in the relationship between low Zn and disease susceptibility are starting to emerge. Plant innate immune mechanisms against biotrophic pathogens are associated with the buildup of SA concentrations, the ROS burst, and the trigger of SA-dependent SAR in non-infected tissues (Cameron et al., 1994; Breitenbach et al., 2014). Different compounds participate in the long-distance transmission of SAR, among them, the C9 dicarboxylic acid, azelaic acid (AzA) (Jung et al., 2009). A recent report revealed an evolutionary conserved Zn sensing-mechanism, which connects root growth to the plant’s pathogen responses (Bouain et al., 2018). The authors found that AZELAIC ACID INDUCED1 [AZI1], a member of the lipid transfer protein family (LTPs) of pathogenesis related (PR) proteins, is triggered by azelaic acid (AzA) during SAR. AZI1 regulated growth and immunity responses depending on Zn availability. The results demonstrated that signaling triggered by low Zn and AzA interact. The low Zn status negatively affected the expression of defense-related genes, among others *PR1*, a SA-induced marker gene that modulated the response to AzA. During early development, when Zn availability is low, *Arabidopsis* can prioritize root growth over defense responses, leading to an increase in soil volume mined for available Zn. The authors highlighted the need for more extensive studies on the plant interacting signaling networks between defense and growth. Along this line, it would be very interesting to test these signaling pathways in genotypes with contrasting Zn efficiency. A close connection between biotic stress defense and growth signaling networks becomes especially evident in the view of the fact that AzA is listed among the agricultural chemicals for plant resistance priming (Aranega-Bou et al., 2014), but was found to severely inhibit root growth in Arabidopsis plants supplied with sufficient Zn concentrations (Bouain et al., 2018).

### Zn-Sequestering Defense Strategies

The requirement of Zn and other micronutrients by pathogens has impelled the animal immune system to evolve mechanisms to sequestrate them in order to restrict pathogen access to these essential nutrients, a process known as nutritional immunity (Hood and Skaar, 2012). There is still little evidence on metal-withholding mechanisms in plants to fight microbes, and most studies focused on Fe. In the case of pathogenic bacteria, the withholding of Fe within a plant storage compartment is considered one of the first mechanisms of defense (Fones and Preston, 2013). A research by Mila et al. (1998) suggested that polyphenols in plants may play a similar role to that of ion-binding proteins in animals by withholding iron away from pathogens. The sequestration of Fe might induce the expression of genes associated with Fe homeostasis and activation of immune responses against biotrophic pathogens (Dellagi et al., 2005). Recently, the expression levels of an apoplastic iron chelating protein has been found to correlate with tolerance to the necrotrophic bacterium *Pectobacterium carotovorum* subsp. *carotovorum* (*Pcc*) (Hsiao et al., 2017).

In the view of the essential role of Zn in key metabolic processes in both plants and pathogens, nutritional immunity mechanisms based on competition for Zn are highly plausible. Metal-chelating compounds could restrict the availability of metals to pathogens in plants. A recent study on the ZINC-INDUCED FACILITATOR [ZIF1], a nicotianamine (NA) transporter located at the tonoplast in *A. thaliana*, showed that the perturbation of the subcellular distribution of the Zn-chelator NA has a great effect in the subcellular and inter-organ partitioning of Zn (Haydon et al., 2012). As suggested by the authors, such studies, in addition to helping to advance the biofortification of crops, also contribute in providing clues concerning Zn-related mechanisms to ameliorate the plant’s resistance to pathogens. Along this line, a *ZIF-1* orthologue in *Zea mays*, *Zm-mfs1*, was previously reported to be induced by fungal infection in resistant as well as in susceptible interactions (Simmons et al., 2003).

### Zn Deficiency-Triggered Defense Mechanisms

Nutrient deficiencies usually render plants more susceptible to pests and pathogens. Nonetheless, some signaling pathways triggered in response to nutrient scarcity boost the plant immune system.

Such a defense activation implying altered gene-expression profiles and further metabolite production has specifically been reported for Fe- and Mn-deficient conditions (Rodríguez-Celma et al., 2013; Fourcroy et al., 2014; Schmid et al., 2014; Rodríguez-Celma et al., 2016). Information regarding Zn deficiency is still scarce, but some studies indicate that pathways signaling Zn-deficient conditions can trigger plant defense responses.

Defensins (DFN) are known for their involvement in the innate immune defense system (Stotz et al., 2009). Recently, an increased expression of three DFN-like proteins in *A. thaliana* grown under Zn-deficient conditions has been found (Inaba et al., 2015). These proteins were under the direct control of the basic region leucine-zipper transcription factor gene *bZIP19*. This transcription factor plays a role in the Zn-depletion responses by regulating ZIP transporter gene expression (Assunçao et al., 2010). The *bZIPs* regulate key processes in both plants and animals. In plants, *bZIPs* participate among other processes, in response to abiotic and biotic stress (Noman et al., 2019a). The bZIP TFs are key players in plant innate immunity regulating genes associated with PAMP-triggered immunity, ET immunity, and hormonal signaling networks (Noman et al., 2017a). Although plant DFN are also implicated in plant growth and development (Stoltz et al., 2009), exploration and functional characterization of novel bZIP TFs *in planta* could be helpful tools to improve crop resistance against pathogens and environmental stresses (Inaba et al., 2015).

## Defense Mechanisms Against Biotic Stress Under High-Zinc Conditions

Many studies have reported enhanced plant resistance to diseases and pests after supplemental Zn concentrations, or when a mix of micronutrients including Zn was applied (Graham and Webb, 1991; Dordas, 2008; Chavez-Dulanto et al., 2018). In fact, several phytosanitary formulae are based on Zn-organic complexes. In this situation, microorganisms and herbivores may be directly affected by the toxic effect of Zn rather than through plant metabolic responses (Graham and Webb, 1991).

### Zn Efflux Defense Strategies

High Zn concentrations are toxic to pests and pathogens. In animals, excess Zn can displace other essential metals from their catalytic center within the protein. Accordingly, the deployment of high Zn concentrations in mucosal surfaces infected with *Streptococcus pneumoniae* has been found to produce a displacement of Mn from the pathogen’s Mn transporters, inducing Mn starvation and enhancing bacterial sensitivity to oxidative stress, among other effects (McDevitt et al., 2011). Few similar Zn efflux mechanisms have been reported in plants, although Stolpe et al. (2017) found that aphid infestation in *Arabidopsis halleri* increased phloem Zn concentration as a first line of defense. Phloem Zn content varied and was higher in the phloem exudates of highly valuable tissues needing protection; i.e., young leaves were better defended than older ones, which agrees with the optimal defense theory (Strauss et al., 2004).

### Zinc-Concentration-Dependent Plant Defense Mechanisms

High Zn concentrations are potentially toxic to all organisms. Zn phytotoxicity usually becomes visible at leaf concentration higher than 300 mg Zn kg^−1^ (Broadley et al., 2007). Zinc toxicity-associated symptoms in plants include reduced yield and stunted growth, reduced export of photoassimilates from leaves to roots (Ruano et al., 1988), and Fe-deficiency-induced chlorosis through reductions in chlorophyll synthesis and chloroplast degradation (Chaney, 1993). The use of high Zn in plant defense depends on the relative host and pest/pathogen Zn-tolerance. In this context, the potential use of Zn as a toxic compound for plant defense has mostly been studied in metal hyperaccumulating species, which tolerate extremely high Zn tissue concentrations. Metal hyperaccumulation can provide defense against herbivores and pathogens in plants, an adaptive advantage that might have driven the evolution of this trait (Boyd and Martens, 1992). Multiple studies have related metal hyperaccumulation to plant defense against biotic stress. Different mechanisms for such metal-based protection have been proposed. According to the original “Metal Defense Hypothesis,” the metal concentrations reached in shoot tissues of hyperaccumulating species would be high enough to intoxicate the attacking pest or pathogen (Boyd and Martens, 1992; Poschenrieder et al., 2006). Afterwards, it was found that high metal concentration and organic compounds may cooperate in the plant’s defense (Boyd, 2012). This led to the “Joint-Effects Hypothesis” proposing that high metal concentrations in non-hyperaccumulating species in cooperation with organic substances can be effective in plant defense. In fact, high Zn concentrations found in non-hyperaccumulating species can enhance plant defense (Cheruiyot et al., 2013; Martos et al., 2016).

### Direct Zn Toxicity

Direct elemental defense by Zn implies that the high Zn accumulation in the plant tissues is more toxic to the pest/pathogen than the plant. A trade-off between metal-based and organic defenses may further benefit the plant as a potential energy-saving mechanism under biotic stress (Strauss et al., 2002). However, no hard data are available comparing the energy costs for production of organic defenses and metal accumulation, chelation, and compartmentation to demonstrate such a trade-off. Few studies have shown a direct Zn toxicity mechanism in plants against pathogens. In the hyperaccumulating species *Noccaea caerulescens*, high tissue Zn concentrations restricted the infection of the biotrophic pathogen *P. syringae* pv. *maculicola*. ROS-dependent defense mechanisms commonly associated with plant defense against biotrophs, such as callose deposition and pathogenesis-related (PR) gene expression, were not triggered despite the fact of increased salicylic acid (SA) levels in the infected plants. This suggests that in this model, a direct toxic effect of Zn would be the main factor limiting bacterial colonization (Fones et al., 2010; Fones and Preston, 2013).

**Figure 2:**
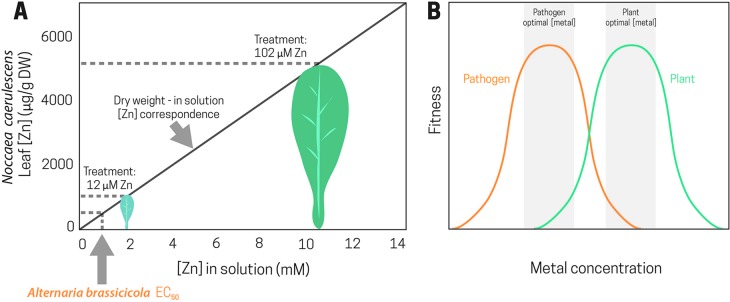
Zinc concentration that caused a 50% inhibition of *Alternaria brassicicola* growth *in vitro* (*Alternaria brassicicola* EC_50_) and theoretical correspondence between Zn leaf concentration and Zn concentration in solution in *Noccaea caerulescens* plants grown at 12 and 102 µM Zn **(A)**. Plant and pathogen response curves to metal concentration. A higher optimal metal concentration for the plant fitness than for the pathogen can lead to protection against diseases by elemental defense **(B)**.

A recent study monitoring the effect of high Zn supply in the response of *N. caerulescens* to *Alternaria brassicicola* (Gallego et al., 2017) showed that the average *Noccaea* leaf Zn concentration, after 5 weeks of growth in 25% modified Hoagland solution with 102 µM ZnSO_4_, was 5,000 µg/g dry weight. This is equivalent to 10 mM Zn in the cell sap, a concentration 20 times higher than the EC_50_ for *A. brassicicola in vitro* (**Figure 2A**). Because *Alternaria* is a necrotrophic fungus, a direct effect of Zn would be feasible due to the release of toxic Zn concentrations into the extracellular space as a consequence of cell compartmentation destruction during the colonization process (**Figure 2B**). However, joint effects may also operate, as in this pathosystem, high Zn also triggered organic defenses.

Nonetheless, most supporting evidence for direct Zn toxicity as a plant defense mechanism in Zn hyperaccumulating species has come from studies with insect herbivores (Boyd, 2007; Vesk and Reichman, 2009). High Zn exposure induced metal accumulation in insect bodies and eggs, causing lower fecundity (Shu et al., 2009, Shu et al., 2012), reduced growth, and higher mortality rates (Nursita et al., 2005; Jhee et al., 2006; Noret et al., 2007; Lagisz, 2008; Kazemi-Dinan et al., 2014). The high Zn concentration in Zn hyperaccumulating species can affect insect herbivores with a chewing feeding mode. High Zn concentrations in the diet resulted in a clear deterrent effect (Behmer et al., 2005; Kazemi-Dinan et al., 2014; Kazemi-Dinan et al., 2015; Stolpe et al., 2017). Fewer studies have focused on insects with a sucking–feeding mode. A Zn-rich diet increased the growth of the generalist aphid *Myzus persicae*, while enrichment with Cd had no effect (Stolpe and Müller, 2016). However, the combination of both metals produced a greater negative effect on the phloem-sucking insects with respect to individual metals. The higher phloem concentrations of Cd and Zn found in the metal hyperaccumulator *A. halleri* growing on metal-rich soils deterred aphid infestation (Stolpe et al., 2017). Furthermore, Zn can additively enhance the toxicity to insects of other metals (Cd, Ni, or Pb). This has been observed both in studies using artificial diets (Jhee et al., 2006) and by adding leaves of hyperaccumulator plants to the insects’ diet (Kazemi-Dinan et al., 2014). A global overview concerning the influence of Zn hyperaccumulation on the plant–predator interaction is given in **Table 2**.

**Table 2:** Overview of the influence of Zn hyperaccumulation on the plant–predator interaction.

Plant species	Metal	Diet	Biotic stress factor (BSF)	Type of BSF	BSF response	Plant protection	References
*Arabidopsis halleri*	Zn-Cd	Natural	*Myzus persicae*	Generalist aphid	Increase GSL in phloem of young leaves	Yes	Stolpe et al., 2017
*Brassica rapa*	Zn-Cd	Artificial	*Myzus persicae*	Generalist aphid	Less survival and performance	Yes	Stolpe and Müller, 2016
*Sinapis alba cv. salva*							
*Arabidopsis halleri*	Zn-Cd	Leaf discs	*Pieris napi*	Crucifer specialist caterpillar	Additive toxic effect	Yes	Kazemi-Dinan et al., 2014
		Leaf discs	*Athalia rosae*	Crucifer specialist sawfly larvae	Deterrence effect		
		Leaf discs	*Phaedon cochleariae*	Crucifer specialist beetle			
		Artificial	*Heliothis virescens*	Generalist caterpillar			
*Arabidopsis halleri*	Zn-Cd	Natural	*not defined*	Leaf-damaging insects	Increase GSL in young leaves	Yes	Kazemi-Dinan et al., 2015
None	Zn / Zn-Cd	Artificial	*Plutella xylostella*	Crucifer specialist moth larvae	Zn enhances the toxicity of Cd	Yes	Jhee et al., 2006
			*DBM larvae*				
*Noccaea caerulescens*	Zn	Natural and	*Schistocerca gregaria*	Polyphagous locust	Post-ingestive rejection of high-Zn diets	Yes	Behmer et al., 2005
		artificial			No difference between diets		
*Noccaea caerulescens*	Zn	Natural	*Schistocerca gregaria*	Polyphagous locust	Post-ingestive rejection of high-Zn plants	Yes	Pollard and Baker, 1997
			*Deroceras caruanae*	Slug	Post-ingestive rejection of high-Zn plants		
			*Pieris brassicae*	Caterpillar	Deterrence effect		
*Noccaea caerulescens*	Zn	Natural	*Pieris napi oleracea*	Caterpillar	Deterrent effect of high-Zn leaves	Yes	Jhee et al., 1999
None	Zn	Artificial	*Plutella xylostella*	Crucifer specialist moth larvae	Toxicity at normal Zn range	Yes	Coleman et al., 2005
None	Zn	Artificial	*Spodoptera exigua*	Generalist larvae	Toxicity at normal Zn range	Yes	Cheruiyot et al., 2013
*Noccaea caerulescens*	Zn	Natural	*Helix aspersa*	Generalist hervibore	Choice of food not affected	No	Noret et al., 2005
*Noccaea caerulescens*	Zn	Natural	*Helix aspersa*	Generalist hervibore	No deterrence effect	No	Noret et al., 2007
*Arabidopsis halleri*	Zn	Natural	*Helix aspersa*	Generalist hervibore	No discrimination due to internal Zn	No	Huitson and Macnair, 2003
*^1^Noccaea praecox*	Cd/Zn	Natural	*^2^Cantareus aspersus*	Generalist hervibore	No Zn protection. Glucose main factor in food choice	No	Llugany et al., 2019

^1^Formerly Thlaspi.

^2^Formerly Helix.

natural diet means plant material, and artificial diet means synthetic diet.

Direct metal toxicity caused by the ingestion of hyperaccumulator plants is difficult to test, as any other potential organic defense may contribute and mask the results. Most studies have been conducted using species belonging to the Brassicaceae family. In these species, glucosinolates (GS) are constitutive or stress-induced compounds that, upon damage, generate hydrolysis products active against insects and pathogens (Hopkins et al., 2009; Kissen et al., 2009).

In order to evaluate the role of Zn in plant defense, the presence of organic defense compounds such GS in Brassicaceae must be considered. For example, Stolpe et al. (2017) conducted a study on the effect of Cd and Zn exposure and aphid infestation on the phloem exudate composition of *A. halleri* and found higher amounts of GS in addition to Zn for Zn-supplemented plants.

Several studies on the feeding preferences of snails with *Thaspi caerulescens* and *A. halleri* and high Zn showed that Zn-hyperaccumulation did not play a key role in leaf palatability, with GS proving more efficient than Zn in deterring these generalist herbivores (Huitson and Macnair, 2003; Noret et al., 2005, Noret et al., 2007). Recently, it has been observed that the snail *Cantareus aspersus* preferentially feeds on leaves of the Zn-Cd-hyperaccumulator *Noccaea praecox* with low metal concentrations (Llugany et al., 2019) and, better yet, low Zn-Cd leaves with the lowest GS concentrations. However, more prevailing for preferred consumption than low metal and GS was a high leaf sugar concentration (Llugany et al., 2019).

### High Zn Triggers Organic Defenses

Besides its potential direct toxic role in defense, high Zn leaf concentrations may contribute to defense by the priming of defense signaling pathways and enhanced structural defenses (Poschenrieder et al., 2006). High metal concentrations and pathogens trigger signaling pathways that share common elements (Mithöfer et al., 2004). Salicylic acid and jasmonates (JA) play pivotal roles in the systemic defense of plants against biotic stress (Thomma et al., 1998). Nguyen et al. (2014) reported that a JA-induced enhancement of plant DFN increased Zn tolerance in the non-hyperaccumulating species *A. thaliana*. Moreover, the Zn-tolerant species, *A. halleri*, constitutively expressed high DFN and showed an increased tolerance to the necrotrophic fungi *Botrytis cinerea*.

**Figure 3:**
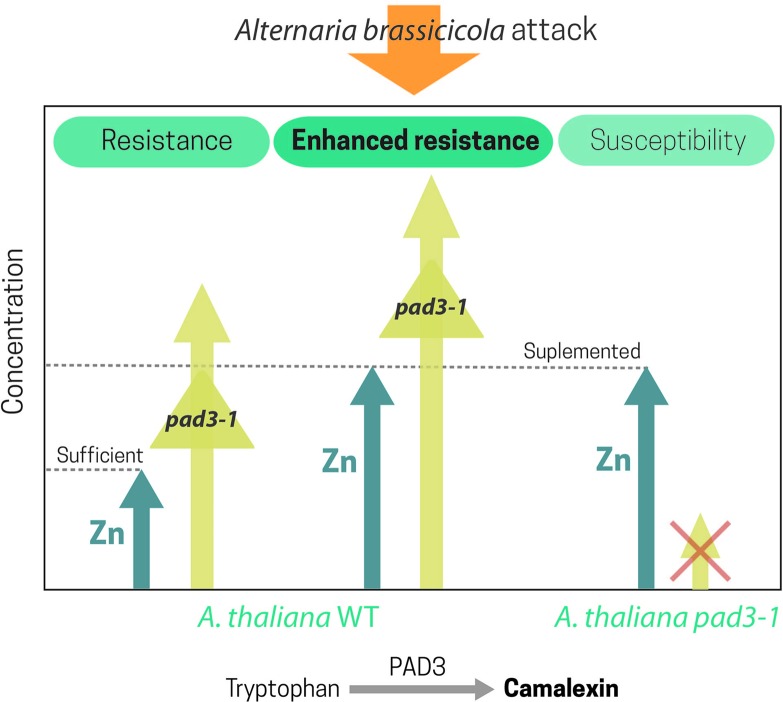
Zn and camalexin, a phytoalexin essential for *A. thaliana* resistance against *Alternaria brassicicola*, showed a joint effect in the Arabidopsis response to *Alternaria*. High leaf Zn concentration could not substitute the role of camalexin in the Arabidopsis camalexin-deficient mutant, *pad3*, infected with *A. brassicicola*. Nonetheless, in the wild type, Zn supplementation greatly enhanced the JA-ET-dependent defense signaling pathway and the expression of PAD3, an enzyme that catalyzes the last step in camalexin synthesis.

It is noteworthy that in plants with normal Zn supply, the xylem-colonizing bacteria *Xylella fastidiosa* requires the expression of Zn detoxification mechanisms for successful host colonization (Navarrete and De La Fuente, 2015). A recent study by Martos et al. (2016) using different non-hyperaccumulating *Arabidopsis* genotypes infected with the necrotrophic fungi *A. brassicicola* highlighted the key importance of Zn in plant defense and supported the joint effect hypothesis (Boyd, 2012), i.e., the cooperation between metal and organic defenses in plants. Camalexin is a phytoalexin that is essential for the *A. thaliana* resistance against *A. brassicicola* (Thomma et al., 1999). Experimental infection with *A. brassicicola* of the camalexin-deficient mutant of *A. thaliana*, *pad3*, revealed that high leaf Zn concentrations could not completely substitute the role of camalexin. However, in the wild type, Zn enhanced the JA-ethylene (ET)-dependent defense signaling pathway and the expression of PAD3, an enzyme that catalyzes the last step in camalexin synthesis (**Figure 3**).

Moreover, Zn hyperaccumulation is essential for *N. caerulescens* resistance to *A. brassicicola* infection. Regardless of the Zn concentration, *Alternaria* triggers JA-ET- and SA-dependent defense pathways. However, only Zn hyperaccumulating plants showed incompatible interactions with the pathogen. Nonetheless, a joint effect of Zn and organic defenses could not be discarded, as higher GS concentrations were also found in *Alternaria*-inoculated Zn hyperaccumulating plants (Gallego et al., 2017).

Nitric oxide (NO) is reported to play a role in the plant response to toxic Zn concentrations (Xu et al., 2010). Recently, NO has been found in *Solanum nigrum* to participate in the expression of many Zn-mediated miRNA, with the predicted target genes indicating that excess Zn modulates pathogen tolerance and the transcriptional process by miRNA pathways (Xie et al., 2017).

## Pathogens and Pests Evolve Mechanisms to Counteract Zinc-Related Plant Defense

To counteract the host-imposed Zn scarcity or toxicity, pathogens and pests have evolved efficient Zn management mechanisms and the capability to use Zn to increase virulence.

In pathogens, high Zn use efficiency implies mechanisms that lead to higher Zn import, export, or/and Zn-buffering capacity. In bacteria, the Zn uptake regulator (Zur), a Zn-responsive transcription factor, member of the Fur family of proteins, is the most common transcription factor that regulates cellular Zn uptake and efflux as well as Zn intracellular trafficking (Choi et al., 2017). Several studies have shown that Zur regulates target genes in a graded manner (Mikhaylina et al., 2018). In the model actinobacteria, *Streptomyces coelicolor*, Zur activates the high-affinity ABC transporter *znuABC*, and its homologue, *znuB2C2* under Zn scarcity. At sufficient Zn levels, the expression of the Zn exporter *zitB* is low, and genes for Zn uptake are repressed. At high Zn, *zitB* induction is also elevated (Choi et al., 2017). This fine regulation of Zn activity allows pathogenic bacteria to maintain virulence over a wide range of Zn concentrations in the extracellular media. In addition, under Zn scarcity, pathogenic bacteria and fungi can use host metalloproteins as sources of metals (Neumann et al., 2017). These mechanisms have been predominately studied in human systems that use Zn-sequestering proteins for defense. These include the neutrophil protein calprotectin (CP) to restrict Zn availability at the infection sites (Striz and Trebichavsky, 2004). Host Zn chelation is a key strategy to cope with infectious process (Ma et al., 2015). However, different pathogenic bacteria have evolved strategies to use host Zn-chelating proteins, including CP, as a source of Zn (Stork et al., 2013; Jean et al., 2016; Neumann et al., 2017).

Zinc detoxification has been reported to be an important virulence factor in bacteria infecting Zn hyperaccumulator species (Fones et al., 2010), as well as non-hyperaccumulating species (Nikraftar et al., 2013). In *X. fastidiosa*-infected plants, the modification of the host leaf ionome correlates with bacterial virulence (Oliver et al., 2014). This xylem-colonizing bacterium has to detoxify Zn for successful host colonization. The knockout of *Zur* genes increases the sensitivity of *X. fastidiosa* to Zn and decreased its virulence (Navarrete and De La Fuente, 2015). Both pathogenic bacteria and fungi modify Zn bioavailability to enhance their virulence. High Zn concentrations induce the enhanced production of exopolysaccharides (EPS), a pathogenic factor in *X. fastidiosa* (Killiny et al., 2013). Enhanced EPS production also indirectly potentiates virulence, as Zn chelation by the negative charges of EPS decreases Zn bioavailability to the bacterial cell (Navarrete and De La Fuente, 2014). Similarly, high Zn concentrations in a lung secretion-mimicking medium enhanced the biofilm formation and virulence of *Pseudomonas aeruginosa* (Marguerettaz et al., 2014).

Fungal Zn homeostasis is regulated by a major transcription factor, the Zn responsive activator protein 1 (Zap1) (Zhao and Eide, 1997). Sufficient intracellular Zn levels are sensed by a direct interaction of the metal and the protein, causing the inhibition of the expression of Zap1 target genes (Frey and Eide, 2011). Under low Zn conditions, pathogenic fungi develop complex metal detection and signaling networks distinct to those deployed by bacteria for Zn uptake (Gerwien et al., 2018). Nonetheless, in response to Zn scarcity, fungi increase the expression of high-affinity membrane transporters to enhance Zn uptake as well as tonoplast Zn transporters that allow Zn storage into the vacuole, the zincosome (Crawford et al., 2018).

Zinc metalloproteins also play a role in the virulence of pathogenic bacteria and fungi. The oxidative burst is an early event in the plant response to diseases (Govrin and Levine, 2000). In some cases, pathogens also use ROS to enlarge their colonization process; therefore, the potential to detoxify ROS is an enhancing trait for the pathogen’s virulence. Accordingly, the reduced virulence of a *B. cinerea* strain defective in the expression of Cu-Zn-SOD in tomato and *Arabidopsis* plants was due to an increase in O_2_^−^ and decreased H_2_O_2_, which was associated with an increase in callose (López-Cruz et al., 2017). On the other hand, pathogenic bacteria have evolved SOD-related mechanisms to evade the host immune system. For example, in the host cells, the withholding of Zn by CP could be expected to decrease the activity of SOD5 in *Candida albicans*. However, to circumvent the decrease in Zn availability, *C. albicans* SOD5 has lost Zn-binding activity and evolved a Cu-only cofactor requirement (Gleason et al., 2014).

Metalloproteases that use Zn as a cofactor are further potential virulence factors during bacterial and fungal infection. Metalloproteases have been reported to suppress the host immune responses in animal systems (Staats et al., 2013; Sun et al., 2016). Zn-metalloproteases, in addition to breaking down the host defensive barriers, could interfere in the host-defense mechanisms. However, their specific role in fighting the plant immune system remains elusive (Figaj et al., 2019).

Herbivores have also elaborated protective mechanisms against the presence of toxic Zn concentrations in plant tissues. The ingestion of toxic metal concentrations induces ROS causing oxidative stress in herbivores (Ihechiluru et al., 2015). In order to survive, herbivores activate an effective antioxidant defense system (enzymes and non-enzyme compounds) that inhibits the metal-induced oxidative damage (Barata et al., 2005). Variations of SOD and catalase (CAT) activities have been observed in several herbivores under metal toxicity stress (Zhang et al., 2011; Wu and Yi, 2015). Apart from antioxidant enzymes, acid phosphatase (ACP) and alkaline phosphatase (AKP) have been associated to the detoxification. An example is the observation of enhanced activity of these metalloenzymes in the lepidopter *Lymantria dispar* when feeding on Zn-treated *Populus* (Jiang and Yan, 2018).

Additionally, herbivores might deal with the presence of high levels of plant secondary metabolites in leaves as a plant defense response to metal accumulation (Huitson and Macnair, 2003; Noret et al., 2005, Noret et al., 2007). Plant secondary metabolites may induce oxidative stress similarly to that caused by heavy metals. Enhancement of glutathione (GSH) production by herbivores has been proposed as a key mechanism to cope with this stress situation (Schlaeppi et al., 2008; Barbehenn and Kochmanski, 2013). A GSH-mediated detoxification process in response to high Zn has recently been reported (Mohr et al., 2018) in a study identifying relevant novel genes that confer Zn sensitivity or tolerance in *Drosophila melanogaster*. The authors found that a gene encoding an ABCC-type transporter family protein, related to yeast Cd factor (YCF1) implicated in the GSH-mediated detoxification of Cd, was up-regulated by excess Zn and conferred tolerance to toxic Zn concentrations in *Drosophila*.

## Conclusions and Future Studies

The multifunctionality of Zn in all living organisms provides this element key roles in basal metabolism, defense, and virulence, turning Zn into a highly valuable tool to understand the mechanisms underlying the plant–pest/pathogen interactions.

The presence of highly efficient Zn sensing probes and Zn detoxification systems in herbivores and pathogens indicates the relevance of Zn-related defense mechanisms in plants, which include different stratagems not restricted to Zn-hyperaccumulating species. Nonetheless, some pests and pathogens have evolved mechanisms to circumvent the plant’s Zn-poisoning strategies. Therefore, future studies should tackle this issue in a more integrative manner by contributing to the understanding of the zinc homeostatic mechanisms in plants and pests and pathogens, as well as to their interaction. Keystones for future research advances should further clarify the double role of defensins as Zn-ligand and biotic stress signaling molecules, the ligand exchange processes, and the cellular and subcellular compartmentation mechanisms for both Zn and its ligands. The outcomes of these studies would strongly contribute to visualize potential strategies that might benefit the host immune system and to design specific Zn-related tools to trick plant attackers or/and to ameliorate plant defense.

## Author Contributions

All coauthors contributed to search of information and structuring of the review. CC and SM wrote the manuscript with inputs from RT, ML, and CP. BG drew the figures and CP acted as the coordinator and the supervisor.

## Funding

This study was funded by the Spanish Government (Ministerio de Ciencia, Innovación y Universidades; projects BFU2013-42839-R and BFU2016-75176-R).

## Conflict of Interest

The authors declare that the research was conducted in the absence of any commercial or financial relationships that could be construed as a potential conflict of interest.
